# Understanding Treatment Needs of Youth in a Remote Intensive Outpatient Program Through Solicited Journals: Quality Improvement Analysis

**DOI:** 10.2196/45509

**Published:** 2023-05-03

**Authors:** Michelle Evans-Chase, Rachel Kornmann, Bethany Peralta, Kate Gliske, Katie Berry, Phyllis Solomon, Caroline Fenkel

**Affiliations:** 1 School of Social Policy and Practice University of Pennsylvania Philadelphia, PA United States; 2 Behavioral Health Care Rutgers University New Brunswick, NJ United States; 3 Charlie Health, Inc Bozeman, MT United States

**Keywords:** youth, young adults, recovery, intensive outpatient programming, telehealth, qualitative methods

## Abstract

**Background:**

Youth experiencing high-acuity mental health symptoms often require highly restrictive levels of care (ie, inpatient care) that removes them from the relationships and activities essential for healthy development. An alternative treatment gaining evidence in its ability to support this population is the intensive outpatient programming (IOP) model. Understanding the experiences of adolescents and young adults *during* IOP treatment episodes may enhance clinical responsiveness to changing needs and protect against transfer to inpatient care.

**Objective:**

The objective of the analysis reported here was to identify heretofore unrecognized treatment needs of adolescents and young adults attending a remote IOP to help the program make clinical and programmatic decisions that increase its ability to support the recovery of program participants.

**Methods:**

Treatment experiences are collected weekly via electronic journals as part of ongoing quality improvement efforts. The journals are used by clinicians proximally to help them identify youth in crisis and distally to help them better understand and respond to the needs and experiences of program participants. Journal entries are downloaded each week, reviewed by program staff for evidence of the need for immediate intervention, and later deidentified and shared with quality improvement partners via monthly uploads to a secure folder. A total of 200 entries were chosen based on inclusion criteria that focused primarily on having at least one entry at 3 specified time points across the treatment episode. Overall, 3 coders analyzed the data using open-coding thematic analysis from an essentialist perspective such that the coders sought to represent the data and thus the essential experience of the youth as closely as possible.

**Results:**

Three themes emerged: *mental health symptoms*, *peer relations*, and *recovery*. The *mental health symptoms* theme was not surprising, given the context within which the journals were completed and the journal instructions asking that they write about how they are feeling. The *peer relations* and *recovery* themes provided novel insight, with entries included in the *peer relations* theme demonstrating the central importance of peer relationships, both within and outside of the therapeutic setting. The entries contained under the *recovery* theme described experience of recovery in terms of increases in function and self-acceptance versus reductions in clinical symptoms.

**Conclusions:**

These findings support the conceptualization of this population as youth with both mental health *and* developmental needs. In addition, these findings suggest that current definitions of recovery may inadvertently miss supporting and documenting treatment gains considered most important to the youth and young adults receiving care. Taken together, youth-serving IOPs may be better positioned to treat youth and assess program impact through the inclusion of functional measures and attention to fundamental tasks of the adolescent and young adult developmental periods.

## Introduction

### Intensive Outpatient Programming

An intensive outpatient program delivered in person [1,2] and remotely [3,4] is an effective modality for treating youth and young adults ranging in age from 11 to 25 years with complex mental health needs. Intensive outpatient programming (IOP) is a model that can serve youth whose needs lie beyond once-weekly outpatient services by providing 9 to 12 hours of treatment per week to youth living at home, allowing them the opportunity to maintain the normative peer relationships and drive for autonomy that are both vital to this developmental period and disrupted by inpatient treatment.

Understanding the experiences of youth with complex mental health needs *during* IOP treatment episodes may further protect against the transition of youth to inpatient care by increasing clinical responsiveness to new or changing needs. In addition, the documentation of these experiences in the client’s own words may reveal needs that are otherwise unidentified when using clinical measures alone [5-8]. The objective of this report was to present a summary of the experiences of youth attending a remote IOP collected through weekly journals as part of ongoing quality improvement (QI) efforts. The goals of the program in collecting these data were (1) to provide program clinicians with a tool to help identify youth who experience crises while in treatment and (2) to identify, understand, and better respond to the needs and experiences of program participants.

### Background

Adolescents and young adults (*youth*) with complex mental health needs include those with multiple or persistent psychiatric or substance use disorders, with co-occurring histories of trauma or with symptoms that severely affect daily life functioning. Although weekly outpatient treatment is often not enough to support the recovery of youth experiencing high-acuity mental health symptoms, more restrictive levels of care, which remove youth from relationships and activities essential to healthy development, may be more intensive than that is needed. A third alternative that is gaining evidence in its ability to support the recovery of youth in this population is IOP.

IOP provides an average of 9 to 12 hours of treatment per week, with services delivered during times that accommodate school schedules for adolescents and work schedules for young adults. Treatment is provided in group, family, and one-on-one sessions and is delivered by an array of clinical professionals, including psychiatrists, psychologists, counselors, and social workers, as well as specialists trained in therapies such as art and recreational therapy [9]. Recent research supports the effectiveness of IOPs for youth in addressing clinical outcomes, including symptom reduction, fewer hospital admissions and crisis events, and improved overall functioning [1,9].

### Remote IOP

More recently, IOPs have begun using a telehealth model to deliver 100% of their programming via Health Insurance Portability and Accountability Act–compliant video software, with patients and families typically attending sessions from home or school. Other than the telehealth delivery modality, weekly engagement in a remote IOP mimics engagement in a traditional in-person IOP. Unlike place-based programs, however, remote IOPs are not dependent on the population of youth in the immediate geography to create treatment groups. The members of a treatment group can be selected with greater specificity based on the intersections of client diagnoses and identities, given that such programs are populated with youth from a wider geographical dispersion and are thus not dependent on the IOP’s local population of youth.

### Responding to Patient Treatment Experiences and Needs

Evidence for the use of telehealth mental health treatment in general and IOPs in particular continues to grow [3,10]. However, such evidence is overwhelmingly collected before and after treatment and is measurement driven, limited primarily to outcomes defined as important by researchers and clinical teams. This approach, although vital to tracking recovery as clinically conceptualized, may not be as appropriate in cases of complex mental health requirements [8] and can neglect changes in needs and outcomes important to the client [5].

The collection of narrative in vivo treatment experiences in the qualitative tradition, such as that used by the IOP whose QI data are reported here, can provide insight into youth’s treatment and recovery experiences not revealed by measurement-based outcome data and help clinicians to respond more effectively to client needs. Previous research using narrative approaches with youth in mental health treatment suggests that there are recovery experiences important to youth that fall outside of common clinical measures, such as changes in family relationships and functioning [5,7], ability to cope, academic and social functionality [5,6], enhanced sense of meaning in life [8], and feelings of being back to their old selves [11].

### Aims and Clinical Implications

The aim of this report was to present the findings of the QI efforts of a remote IOP serving youth and young adults. The QI analysis presented here sought to summarize the narrative experiences of clients during treatment as a way to better understand their treatment needs. The goal of the program’s ongoing QI efforts is to use journal entries to inform clinical and programmatic decisions that will enable the program to better serve the needs of all youth participating in the program.

## Methods

Narrative data are collected weekly as part of ongoing QI procedures and used proximally to help clinicians identify youth in crisis, with submissions scanned by hand and a text-reading program to identify entries considered high risk related to self-harm, suicidality, or abuse. The journals are also used distally, as reported here, to better understand and respond to the needs and experiences of program participants.

### Ethical Considerations

This project was reviewed and determined by the institutional review board of the University of Pennsylvania to qualify as QI, indicating that these activities are not human subject research requiring an informed consent process.

### Program Characteristics

Charlie Health, Inc, is an intensive outpatient program that provides treatment to adolescents and young adults with complex mental health needs across the United States using a 100% remote platform. The program specializes in treating youth with severe trauma, neglect, or foster care experience; from marginalized gender and sexual minority communities; who reside in rural or native communities; and who have had multiple crisis events in the previous year leading to recurring admissions to more restrictive levels of care settings.

Each incoming client participates in a biopsychosocial intake interview upon which a diagnosis is made and a treatment plan is developed that specifically addresses their diagnosis and unique developmental and psychosocial needs, including needs that arise from membership in minoritized gender and sexual orientation populations. Clients are prescribed 9 to 12 hours of treatment each week, including approximately 9 hours of group therapy, 1 hour of individual therapy, and 1 hour of family therapy. Although the groups are not cohort-based, clients are placed into groups with peers who are similar to them in age, gender, and sexual orientation.

Each day of group therapy includes three 50-minute blocks of evidence-based skill-building interventions (ie, dialectical behavior therapy [DBT] or cognitive behavior therapy), general therapeutic processing, and experiential therapy (ie, art, music, or journaling). Clients also attend individual and family therapy sessions each week with master’s-level licensed clinicians. Finally, psychoeducation and mutual aid groups are offered to parents and others involved in the client’s care, including weekly *IOP road map(s)*, that keep family members up to date on the skills their children are learning that week, along with tips on how to support them in practicing these skills at home.

### Data Collection Procedures

Charlie Health collects data on both measurement-based outcomes (ie, depression via the Patient Health Questionnaire-9 and anxiety via the Generalized Anxiety Disorder-7 questionnaire) and narrative experiences (via solicited journals) as part of ongoing QI efforts. Requests for journal entries are sent weekly via email and text messages to all youth and young adults attending Charlie Health.

#### Consent

Although client consent to use QI data for this purpose is not required given the QI status, it was deemed important that clients had individual agency in the way their journal information was used. Therefore, the introductory statement for the trial journal includes wording regarding the ways in which the information will be used and contact information for a person at Charlie Health should the clients have any questions. Once a journal entry is submitted, a final page is displayed on which clients are thanked for their time and reminded that they will always have the last say in how their information is used and that they can remove their entries from analysis should they ever change their minds. The same contact information is again provided.

In recognition that consent is a process and given the personal nature of individual quotes, all clients whose entries were identified as exemplars of themes to be used in internal reports and outside publications were contacted directly via email for permission to use their entry. Each request included the specific quote proposed for use, an explanation of how the quote would be used, and the assurance that allowing the use of their quote was voluntary with no repercussions should they decide not to provide permission. A copy of this report, with all proposed quotes removed, was provided for context.

#### Solicited Journaling

Solicited journaling is an effective way to collect ongoing narratives of day-to-day experiences [12] that does not rely on participant recall or a point-in-time summation of complex, longitudinal experiences. However, there are challenges to collecting experiences via solicited journals that include encouraging entries that are neither too broad to be summarized nor so narrow as to be uninformative [12]. To address these challenges, Charlie Health uses 2 variations of a web-based journal that provide prompts and instructions to guide youth in knowing what experiences to write about, while at the same time giving room for youth to focus on those parts of the experiences that they find most important (Textboxes 1 and 2; a link to the ongoing version [Textbox 2] is emailed to clients each Monday, with a reminder sent on Thursdays) [12]. The introductory journal is distributed electronically to each new client as part of the intake process in a remote breakout room before their first treatment session. The clients are told that the reason for the journal is to give them a chance to let program staff know how they are doing, what they like and do not like about the program, and whatever else they want the program staff to know. It is made clear that submitting the journals is voluntary.

**Instructions and prompts (version 1, introductory week 1 only).**

- Instructions: Welcome to journaling! This activity will give you a chance to let us know in your own words how you are doing—how things are going for you in general and how they are going for you here at Charlie Health. You can write about any random stuff that you want to—it doesn’t even have to be about your treatment or Charlie Health but feel free to talk about the symptoms of your mental health issues. You can write in any style—paragraphs, sentences, bullet points, or whatever. You can write your own thoughts; you can use quotes from books, movies, or other people; you can use song lyrics, poetry...
- Prompts: Have you ever been to group therapy before? If you have, tell us about your experience. How are you feeling about your first group at Charlie Health? What would you like to get out of group therapy? Is there anything else about yourself or your life that you would like us to know? You can write about any random stuff that you want to.

**Instructions and prompts (version 2, ongoing).**

- Instructions: Welcome to journaling! This activity will give you a chance to let us know in your own words how you are doing—how things are going for you in general and how they are going for you here at Charlie Health.
- Prompts: Tell us about your week. You can write about any random stuff that you want to—it doesn’t even have to be about your treatment or Charlie Health but feel free to talk about the symptoms of your mental health issues. You can write in any style—paragraphs, sentences, bullet points, or whatever. You can write your own thoughts; you can use quotes from books, movies, or other people; you can use song lyrics, poetry...What did you like about Charlie Health this week? What didn’t you like about Charlie Health this week? How can we make Charlie Health better?

### Data Preparation and Analysis

Although most new clients complete the initial journal, with >1000 completed to date, not all youth continue to submit entries throughout their time in treatment. As the distal QI goal for the journals is to better understand the client experience throughout the treatment process, inclusion in this analysis was limited to journals with at least 1 entry at each of 3 time points: *time 1*=introductory journal, *time 2*=entries submitted during the fifth or sixth week of treatment, and *time 3*=entries submitted during the 11th or 12th week of treatment. On the basis of these criteria, 66 journals with 200 entries (*time 1*=66/200, 33%; *time 2*=68/200, 34%; and *time 3*=66/200, 33%) were identified and included in this analysis. These time points were chosen to reflect early, middle, and later points in the treatment experience and were based on the average number of weeks in treatment in the program; for instance, the average number of weeks of treatment ranged from 10 to 12 weeks. The middle point was identified numerically as halfway between week 1 and weeks 10 to 12. As clients are on unique treatment tracks, the assumption going into the analysis was that each treatment point did not necessarily reflect a common therapeutic content at that point in time but would reflect a rough estimation of the amount of therapy experienced up until that point.

Open-coding thematic analysis was used [13,14] from an essentialist perspective, which attempts to summarize the participants’ day-to-day experiences as closely as possible and interpret meanings from the perspective of the participants [15]. In recognition that such analysis is an interplay between the direct perspectives of the clients as communicated in their written words and the biases brought to the reading and interpretation of these words [15], an iterative process with multiple coders was used to protect against the bias of a single interpreter. The first and second authors coded an initial 31 journals (93 entries) individually and then as a team to identify possible codes and themes. The second author then created a codebook in which preliminary codes were defined and examples provided. The second and third authors then used the codebook to code all 200 entries while being open to the possibility of new codes should they arise (none did). Once all entries were coded, the 3 authors met to identify possible sources of bias and differences in the understanding and application of the codes. As a result, code definitions were again adjusted, and the 200 entries were recoded individually by each of the 3 authors and compared, with discrepancies discussed until consensus was reached. The authors recognize that interpretations leading to themes are likely biased by the clinical outcome perspective brought to the QI effort. However, the 3 authors were aware of this bias and intentionally sought to identify outcomes and experiences that fell outside of those clinical outcomes traditionally seen in QI data.

Comparing the 3 time points in terms of differences in themes was considered, given the possible insight that could be obtained into how experiences change across the treatment episode. However, this was ultimately rejected, given that for such comparisons to be valid and thus informative to the program, a more quantitative approach involving significance testing would be required to uncover true changes versus those based on random variation. Such exploration was decided to be outside the scope, methods, and goals of the QI analysis upon which this report is based. Therefore, codes from the 3 time points were combined, with the themes presented in the following sections reflecting those parts of the therapeutic experience that remained predominant across the 12 weeks of treatment.

## Results

### Demographic Characteristics

Entries from 66 clients were included in analysis. Clients ranged in age from 11 to 30 years, with an average age of 16.5 (SD 5.05) years. They attended an average of 15 (SD 4.88) weeks and an average of 42 (SD 15.16) sessions. As summarized in Table 1, 50% (27/54) of the clients who reported their gender were female, 24% (13/54) were male, and 26% (14/54) were gender expansive (genderqueer, gender nonconforming, gender fluid, gender questioning, gender neutral, or nonbinary). Of the 54 clients who reported their sexual orientation, 39 (72%) identified as a member of a lesbian, gay, bisexual, transgender, queer, asexual, intersex, and similar minoritized communities, and 15 (28%) identified as heterosexual or straight. The most common diagnoses were depression (48/66, 73%) and anxiety (37/66, 56%).

**Table 1:** Demographic and clinical characteristics of clients.

Characteristics		Values, n (%)
**Gender (n=54)**		
	Female	27 (50)
	Genderqueer or gender nonconforming	2 (4)
	Gender fluid	3 (6)
	Gender questioning	1 (2)
	Gender neutral	1 (2)
	Male	13 (24)
	Nonbinary	7 (13)
**Sexual orientation (n=54)**		
	Asexual or gray sexuality	1 (2)
	Bisexual	12 (22)
	Gay	5 (9)
	Heterosexual or straight	15 (28)
	Lesbian	5 (9)
	Pansexual	10 (19)
	Queer	1 (2)
	Questioning	5 (9)
**Diagnosis^a^ (N=66)**		
	Depression	48 (73)
	Anxiety	37 (56)
	Cluster B personality	2 (3)
	Conduct or disruptive or oppositional disorder	6 (9)
	Gender dysphoria	3 (5)
	Eating disorder	1 (2)
	Bipolar disorder	1 (2)
	ADHD^b^	4 (6)

^a^Total reflects multiple diagnoses for some of the clients.

^b^ADHD: attention-deficit/hyperactivity disorder.

### Themes and Codes

Table 2 summarizes the themes and associated codes that arose during analysis, along with the frequency with which each was identified across the 200 entries. The 2 most common themes that youth wrote about were peer relations (121/200, 60.5%) and mental health symptoms (122/200, 61%). The third theme, recovery, although seen in only 36.5% (73/200) of the entries, was deemed important enough to include based on the QI goals of the analysis.

**Table 2:** Summary of themes and codes.

Theme		Values, n (%)
**Peer relations (n=121)**		
	Conflict	16 (13.2)
	Support	25 (20.7)
	Wanting engagement	21 (17.4)
	Engagement	59 (48.8)
**Mental health symptoms (n=122)**		
	Objective descriptions	53 (43.4)
	Subjective experiences	69 (56.6)
**Recovery (n=73)**		
	Reduced symptoms	17 (23.3)
	Active coping	24 (32.9)
	Increased capacity	32 (43.8)

### Peer Relations

#### Overview

Peers were defined by the authors as other youth within and outside of the program who were of similar age and with whom no relational hierarchy presently existed. IOP peer facilitators were thus not included as peers under this theme. The codes that together make up this theme are peer conflict, peer support, and the desire for or experiences of positive engagement with peers.

#### Peer Conflict

Youth described conflict with friends and youth within and outside of the treatment setting. Descriptions of conflict with peers outside of the treatment setting ranged from everyday arguments that did not seem to have much of an emotional impact on the writer to conflict that was interpreted as being more emotionally impactful, with one youth writing as follows:

I know this is just a journal to see how I’m doing, but I just HAVE to complain about this person in my group named [XX]. They’re just,,, so annoying????

Another youth made the following journal entry: “I got missgendered a lot by fellow member...”

#### Peer Support

Youth also wrote about the support they received from individual peers inside and outside of the treatment setting as well as support from their treatment group as a whole; for example, one youth described the support they received from friends within the treatment setting:

I am still struggling with feelings of guilt and depression but I am working on myself. Everyone tells me it’s not gonna be this way forever and I want to believe them. I want a better story. I don’t want to live like this forever. To be quite honest I’m miserable. But my support system has hope for me. I want to have more hope for me too.

An example of youth receiving support from their peers within the treatment setting included “I liked how all my group members help each other out even after group sessions.”

#### Peer Engagement

Peer engagement was the most common code under this theme, with 48.9% (59/121) of the entries including commentary about engaging with peers, either in terms of wanting more engagement or descriptions of positive engagement with peers. Wanting more peer engagement was expressed in terms of wanting to make new friends or interact with, and get support from, peers who they felt were similar to themselves; for instance, one youth wrote as follows:

I’m looking forward to seeing other people but nervous about sharing and feeling comfortable with that. [What I want out of CH: to know that not alone and people to connect with around my age where we can talk about our similar experiences.

Many entries described positive engagement with peers inside the treatment setting, with one youth writing as follows:

Group and individual was amazing this last week. I feel really well bonded with my group.

Entries describing missing peers who, for instance, finished treatment ahead of them were also interpreted in terms of positive engagement, with a youth making the following journal entry:

This week has been fine. I’m actually sad because a couple of my favorite group members got discharged (I’m very happy for them though!!) so now it’ll be kind of weird without them.

### Mental Health Symptoms

Unsurprisingly, many of the entries (122/200, 60.5%) addressed mental health symptoms, with some of the youths (53/121, 43.8%) describing symptoms objectively and others (69/121, 57%) adding their subjective experiences in response to them; for instance, one youth’s objective description was as follows:

*I have intrusive “back-of-the-head” suicidal thoughts daily and have had these since my depression began in middle school (mid 2000’s).* *Most of the time I am able to keep these thoughts in check but when I get overwhelmed or extremely depressed.*

An example of a more subjective experience is this journal entry from a youth:

I am nervous to see my friends again. I don’t want to put anything on them or make them feel like they need to be responsible for me...I felt like I constantly needed to feel pain because I gave pain to others so I self harmed. I have had extreme anxiety and depression. I feel safe at home but I am scared to sleep or do much because of voices, panic attacks, and nightmares.

### Recovery

The final theme, *recovery,* encompasses descriptions by youth of positive therapeutic and personal changes that occurred during the time they were in treatment or related directly to the therapeutic process. The codes that make up this theme include reduced symptoms, active coping, and increased capacity.

#### Reduced Symptoms

Entries that described reductions in specific mental health symptoms as well as those that described general feelings of doing better with regard to their symptoms were included in the code *reduced symptoms*; for instance, one youth wrote as follows:

I’m still struggling a little bit with motivation and my overall feelings of depression but these past few days they feel like they’re passing. I finally have hope for the first time in a while and it feels good. Even just a little bit of hope is better than none:).

Another youth wrote more generally:

I feel like I’ve been good! I feel I’ve been consistently in a stable mood, again working a lot.

#### Active Coping

Entries were coded as *active coping* when they described the use of general or specific therapeutic or mental wellness practices outside of the treatment setting; for instance, a youth made the following journal entry:

I was a little tired but made sure to keep myself busy so I didn’t succumb to depression. I actually read my favorite book (A Wrinkle in Time) and it reminded me of the different ways I would cope as a child.

Other youth wrote about practicing specific coping skills that they learned during treatment, such as mindfulness, DBT principles, and yoga; for instance, one youth wrote as follows:

Weeks been okay. Ups and downs but working on my DBT skills, I like Saturday a lot with the art therapy.

#### Increased Capacity

The final code in the *recovery* theme, *increased capacity*, includes both general statements that suggest that the client is doing better or moving forward as well as commentary regarding specific increases in the ability to function; for instance, one youth described in more general terms an experience reflective of increases in inter- and intrapersonal capacity:

I feel exhaustedddd today holy. But also a huge relief has been lifted off me. I talked to my friend who I ended my friendship with over the summer when I was manic. I explained everything and my journey with mental health and what I’ve been going through. She was really understanding and we talked like old times. I’m excited that it feels like me and her have a fresh start and get to move forward with a healthier and stronger friendship. I was really anxious yesterday before talking to her but now a weight has been lifted.

Increases in capacity that were described in specific terms were further explored and delineated to more clearly illustrate how clients conceptualize positive change and to identify important aspects of recovery to these youth. Three primary types of increases were identified: role functioning, therapeutic progress, and self-actualization. Role functioning reflected activities such as going back to work or school, participating more fully and positively in family relations, and increased productivity (eg, shopping and meal preparation). Therapeutic progress reflected the use of coping strategies, communicating more confidently and openly about problems, and participating more in group or individual therapy. Self-actualization reflected self-acceptance and an increase in youth’s confidence to live authentically in who they are. Textbox 3 summarizes the frequency of each capacity alongside exemplar quotes for role functioning and therapeutic process. As self-actualization primarily included discussion of gender and sexual orientation identities that were very personal in nature, extra care was taken to identify exemplar quotes of clients who were aged ≥18 years and clearly *out* to family and friends to reduce the possibility of causing harm, including in the process of asking for permission to use the quotes for this report. Only 2 quotes were identified for which the authors felt that it would be safe to contact the former clients for permission, but neither responded to the request. Therefore, to best protect the identities of those moving toward self-actualization, a summary of examples, rather than direct quotes, has been provided.

**Client-defined increases in capacity (n=32).**

Types, n (%), and quotes

- Role functioning, 25 (78%)
  “I’ve also been doing things to have progress in my life, I got a nice babysitting/overnight gig that will hopefully go on for a while, and I had an interview for a nanny job and I am meeting them in person tomorrow. School has been a helpful distraction that I can focus on more than less.”
  “The week was surprisingly good, with some pitfalls. For one, I was able to finish the book I was reading about boundaries...I started a new book on productivity...I also took the kids outside on a beautiful November day to enjoy our backyard, jumped on the trampoline with them, swung on the swings, and listened to music.”
- Therapeutic progress, 11 (34%)
  “If I were to tell myself from a few months ago how much we’ve changed, I’m not even sure that she’d believe me. It gives a hope that I haven’t had in a long time and I couldn’t be more grateful for the family I created here. And with everything I’ve learnt, I’ve also been able to tell my friends who are also going through hard times some of the things I’ve learnt and they really liked them!”
  “This week has been hard. But I am proud of myself today...Today I used one of my coping skills: I did yoga. I haven’t done yoga in so long so it felt really good that I had motivation for that. And me and my mom have started walking each night so that’s good too. I am still struggling with feelings of guilt and depression but I am working on myself.”
- Self-actualization, 9 (28%)
  Clients came out as nongender binary or to friends or family, changed appearance to better reflect their identity, and made statements about feeling more confident.

## Discussion

### Principal Findings

The aims of the QI analysis reported here were to identify yet unknown perspectives and experiences of youth attending a remote IOP that could inform programmatic and treatment decisions. Although *mental health symptoms* was a common theme, it was elicited by the journal prompts for use proximally as ongoing feedback about client symptoms that may need immediate clinical response. The focus of this section therefore will be on the 2 themes that arose as part of more spontaneous communication and provided the novel perspectives and experiences identified in the QI goals.

The first theme identified across journal entries that provided novel insights with valuable implications for clinical practice with youth was the importance to youth of peer relationships both within and outside of the IOP setting. The second theme concerned the way in which youth experience *recovery*, which they described more in terms of increases in their ability to return to *normal life* than in terms of reductions in clinical symptoms.

Attention to these viewpoints in treatment and clinical services may help organizations to better engage youth in the treatment process and support additional facets of client recovery; for instance, programming that supports youth in navigating healthy peer relations outside of treatment and provides therapeutic space for peer support within treatment may encourage youth to participate more fully in the therapeutic process. In addition, broadening the definitions of recovery beyond reductions in clinical symptoms to include functional increases may help organizations to track improvements not currently seen, increase the responsivity of treatment, and motivate youth by attending to those outcomes that they experience as being important aspects of their recovery along with those defined clinically. This is consistent with the use of the term *personal recovery* rather than *clinical recovery* in the adult mental health treatment arena and is also consistent with person-centered care [16].

These viewpoints can also inform treatment and clinical decisions via the attention they draw to the developmental needs and milestones that are critical to the adolescent and young adult periods. The focus on peer relations seen in the journals, for instance, reflects the development of peer centrality, a primary feature of adolescence, whereas the functional increases that defined youths’ experience of recovery reflect the developmentally normative drive for autonomy and identity development in older adolescence and young adulthood.

### Peer Relations

As mentioned in the *Results* section, the most common theme found across the 200 journal entries addressed youths’ relationships with their peers. Youth described peer conflict; feeling supported by, and wanting to engage with, peers; and having positive experiences with them, within and outside of the treatment setting. Such experiences reflect what is termed peer centrality in the developmental field, a normative developmental process wherein youth rely heavily on their peers for social feedback [17]. These peer relationships are both developmentally vital and unique in that they do not occur in the social hierarchy common to relationships with adults and authority figures. As such, peer relationships offer reciprocity of trust and intimacy as well as conflict and self-reflection on social standing [18].

The peer-centered theme identified here is a developmental marker for all youth. Their emphasis in these journal entries suggests that these relationships are similarly important for youth with high-acuity mental health issues. Of note, the predominant focus for the youth in this analysis was positive peer relations, with journal entries describing the desire for more peer engagement, descriptions of positive peer engagement, and instances of peer support outweighing entries describing peer conflict. This suggests a partiality toward the benefits of peer relationships, even among youth experiencing substantial clinical symptoms, and highlights aspects of life of central importance to youth, regardless of the complexity of their mental health needs or their status as clients in an IOP.

### Recovery

The dominant definition of recovery from a clinical outcome perspective often focuses on symptom reduction. Although youth whose narratives were interpreted in the analysis reported here did describe symptom reduction as clinically defined, these descriptions represented a smaller percentage of what could be interpreted as recovery experiences. By contrast, nearly a third of the entries reflected positive changes within, or in response to, the treatment episode. This suggests that youth were more likely to experience recovery as an increase in their ability to fulfill everyday role obligations, to participate more fully in the therapeutic setting, or to embrace their emerging identities and live a more authentic life. Similar to the concept of personal (vs clinical) recovery in the adult mental health literature, youth’s description of recovery was about achieving their own goals and having hope in spite of continuing to deal with the symptoms of mental illness [19].

These definitions of recovery can also be understood within the normative developmental drive toward increased autonomy as young people broaden their social circles and acquire new competencies. Failure to meet developmental tasks associated with autonomy can result in a host of adverse outcomes throughout the life course [20]. The youth represented here saw therapeutic gains in terms of functional improvement and increases in their ability to cope independently with adversity within and outside of the treatment setting, both of which are reflective of developmentally appropriate increases in autonomy. In addition, the drive for autonomy can be seen in the identity work [21,22] described by the youth that culminated in increased self-acceptance and changes in self-presentation. Youth began representing themselves in a way that more closely paralleled their own identity, often despite, or in conflict with, family and societal expectations. Overall, these findings lend support to autonomy as an important factor in defining and supporting the recovery of youth experiencing high-acuity mental health symptoms.

### Implications

The findings of this thematic analysis present several implications for clinical practice. First, these findings support the conceptualization of youth in mental health treatment as youth with mental health *and* developmental needs. Programs for youth could factor normative developmental tasks of the adolescent and young adult periods (autonomy, peer centrality, and identity formation) into QI outcome decisions and provide programing that explicitly supports youth in their development of healthy peer relationships, increases in autonomy, and navigation of identity consolidation and expression within the context of their mental health needs.

Second, these results support the use of solicited journaling as a valuable tool in QI efforts. Ongoing collection and review of narrative entries can guide programmatic and clinical changes in real time to address the immediate needs of clients as entries are submitted, which is essential for QI. Open-ended prompts allow youth to communicate needs that are outside of clinical expectations, which can help programs improve quality of care for clients in both the short and long term.

### Strengths and Limitations

A considerable strength of this QI analysis is the insight into the mental health treatment experience of youth it provides using youth’s own words. Solicited journaling as a clinical tool avoids point-in-time recall bias inherent in most postexperience measurement tools, allowing for an investigation of what youth find to be most salient during early recovery. One primary limitation should also be considered. Given that the goal of qualitative inquiry is to provide a richer understanding of various aspects of the experience of a set of individuals [23], the findings of this investigation may not be generalizable to a broader population. In fact, the goal of this QI analysis was to inform treatment considerations for the remote IOP and not to generalize beyond the youth participating in the program. However, this report was written with the recognition that although the youth whose entries were used here do not represent all youth in an IOP, they are not so unique that the findings cannot inform other similar settings and populations of youth [24].

### Conclusions

A principal conclusion that can be drawn based on this analysis is that current definitions of recovery by clinicians and systems of care may inadvertently miss supporting and documenting treatment gains considered most important to the youth and young adults receiving care. As evidenced by the journals used in this analysis, youth are calling for greater attention to their ability to fulfill responsibilities and social obligations while still learning to navigate life *along with* their complex mental health needs—evidence of an increased ability to use adaptive coping mechanisms in the face of everyday life stress. As such, youth-serving IOPs may be better positioned to serve youth and assess program impact through the inclusion of functional measures and attention to fundamental tasks of the adolescent and young adult developmental periods. Symptom improvement does not necessarily coincide with social or functional improvements [25], suggesting a greater need for attention to such outcomes. The added value of measuring functional improvements is that gains in this domain may provide a fuller narrative [26] of both personal and clinical recovery by demonstrating how functional gains may differ in consistency from the ebb and flow of clinical symptoms as youth encounter life stressors during and after participation in an IOP; for instance, although youth may have increases in clinical symptoms of anxiety in response to a particularly stressful life change, their ability to attend school or socialize with friends may remain stable.

Finally, this investigation is but an indication of what is likely a wealth of untapped information about how youth recover and what their unique journeys look like throughout the course of treatment. Recognition of this discrepancy between conceptualizations of improvement after high-acuity care as defined by systems of care (ie, clinical symptom reduction) and what youth themselves see as important (peer relations, autonomy, and self-acceptance) is an important step in moving the field forward to better serve this high-need population. Continuing to weave in personal narratives to better understand the larger narrative of change predominantly told with quantitative outcomes permits opportunities to affect additional positive change in youth that may have been previously missed.

## Abbreviations

DBT: dialectical behavior therapy
IOP: intensive outpatient programming
QI: quality improvement

## Data Availability

The data used for this report were collected by program staff as part of the clinical process and for administrative and ongoing quality improvement efforts. Therefore, the data are not available for public use.
